# Knowledge, Attitudes, and Perceptions of Maternity Care Providers on the Implementation of Calcium Supplementation during Pregnancy in Three Public Hospitals in Argentina: A Qualitative Study

**DOI:** 10.3390/nu16162734

**Published:** 2024-08-16

**Authors:** Martín Hernán Di Marco, Wanda Cabrera, Tomas I. Rivas, Eduard Maury-Sintjago, María N. López, Gabriela Cormick

**Affiliations:** 1 Department of Criminology and Sociology of Law, Faculty of Law, University of Oslo, 0130 Oslo, Norway; m.h.d.marco@jus.uio.no; 2 Universidad Nacional de La Matanza, San Justo 1754, Argentina; wandadenisse.c@gmail.com (W.C.); rivastomas184@gmail.com (T.I.R.); 3 Department of Nutrition and Public Health, University of Bio-Bio, Chillán 3780000, Chile; emaury@ubiobio.cl; 4 Centro de Investigaciones en Epidemiología y Salud Pública (CIESP), Consejo Nacional de Investigaciones Científicas y Técnicas (CONICET), Buenos Aires 1414, Argentina; 5 Universidad Nacional José Clemente Paz, José C. Paz 1665, Argentina; mar_ln90@live.com; 6 Instituto de Efectividad Clínica y Sanitaria (IECS), Consejo Nacional de Investigaciones Científicas y Técnicas (CONICET), Buenos Aires 1414, Argentina

**Keywords:** calcium, supplementation, health-care providers, perceptions, pregnancy, implementation, preeclampsia, Argentina

## Abstract

The aim of this study was to explore maternity care providers’ knowledge, attitudes, and perceptions about the use of calcium supplements during pregnancy for the prevention of preeclampsia in three hospitals from Metropolitan Buenos Aires, Argentina. We conducted semi-structured interviews and followed a thematic analysis framework. Maternity care providers’ knowledge, attitudes, and practices regarding calcium supplementation during pregnancy are linked to barriers to the potential implementation of calcium supplementation. Free provision of calcium supplements by the government, coupled with training that reinforces the scientific evidence supporting their use to prevent preeclampsia, along with documented recommendations from credible sources, would be crucial to ensure that health providers adopt the use of calcium supplements in antenatal care. Future studies should assess pregnant women and policymakers’ perceptions about calcium supplementation during pregnancy, as well as local infrastructure to provide access to free-of-charge calcium supplements in antenatal care settings. Economic evaluation with local information could inform policymakers and advocate for the implementation of strategies to reduce preeclampsia.

## 1. Introduction

Prenatal care is essential to protect pregnant women’s and newborns’ health [1,2]. As a fundamental way to access the health system, prenatal care is an opportunity for the prevention, detection, and treatment of prevalent diseases [3]. Hypertensive disorders of pregnancy (HDP) are among the leading obstetric causes of maternal mortality worldwide [4]. HDP cause around 50,000 maternal deaths and 500,000 neonatal deaths annually worldwide [4]. Calcium supplementation reduces the occurrence of HDP and halves the occurrence of preeclampsia in populations with low calcium intake. Prevention is the most affordable and cost-effective approach for communities [5].

The World Health Organization (WHO) recommends calcium supplementation from the 20th week of gestation for the prevention of preeclampsia [6]. However, the translation of WHO recommendations into national guidelines, policies, and clinical practices has been slow. Consequently, rates of preeclampsia are not declining in regions where calcium supplementation is recommended [7].

A recent review highlights that barriers to pregnant women taking calcium supplements were limited knowledge about calcium supplements and preeclampsia, fears of side effects, and the burden of taking more supplements, while maternity care providers felt adequate staff, training, and calcium supply would help [8]. The review also reports that users and maternity care providers agreed that receiving information regarding preeclampsia and ensuring supplement safety would facilitate the implementation [8]. Studies included in the review were mainly from sub-Saharan Africa and Asia and none from Latin America [8].

In Argentina, as in many low- and middle-income countries, access to calcium-rich foods is insufficient to meet nutritional requirements [9,10]. Calcium is one of the micronutrients with high prevalence of inadequate intake in Argentina [11]. Local authorities are evaluating the inclusion of calcium supplementation in pregnancy care guidelines and recommendations. This evaluation comes as maternal mortality has increased in the country since 2015, with HDP being identified as the main cause [6,12]. Formative research could help identify context-specific barriers that can be addressed to facilitate the implementation of calcium supplementation during pregnancy in Argentina [13].

The aim of this study was to explore maternity care providers’ knowledge, attitudes, and perceptions about the use of calcium supplements during pregnancy for the prevention of preeclampsia in three hospitals in Metropolitan Buenos Aires.

## 2. Materials and Methods

In this qualitative study, we investigated the knowledge, attitudes, and perceptions of calcium supplementation during pregnancy among maternity care providers from three hospitals of the public sector located in Metropolitan Buenos Aires, Argentina. The health-care system in Argentina comprises the public, private, and social security sectors. Approximately 60% of births occur in the public sector and 98% of births are assisted by trained maternity care providers [12,13]. The population in the public sector is fully covered by the state and has no free choice of health-care provider for prenatal care or childbirth.

Two of the hospitals, Governor Domingo Mercante (Mercante Hospital) and Mariano and Luciano de la Vega (Moreno Hospital), belong to the province of Buenos Aires hospital network and one, Ostaciana B. de Lavignolle Hospital (Morón Hospital), is a municipal hospital located in Morón.

Mercante Hospital provides coverage to a population of approximately 1,400,000 inhabitants, of which 40% are covered by the public sector. The hospital has around 3800 deliveries a year and has a total of 19 obstetricians and gynecologists, 30 midwifes, and 16 nurses linked to the service. Moreno Hospital has around 2600 deliveries a year and has 15 obstetricians and gynecologists, 30 midwifes, and 20 nurses working for the service. Morón Hospital has around 2339 deliveries a year and has 26 obstetricians and gynecologists, 21 midwifes, and 16 nurses working for the service.

Maternity care providers were selected if they worked in any of these hospitals providing antenatal care. However, we did not aim to assess differences in the perceptions of the interviewed maternity care providers (e.g., different responses between nurses and physicians). They were invited to participate using a quota sampling technique to represent the views of different professions, genders, and age groups. All maternity care providers willing to participate had the objective of the study and the duration of the interview explained to them. Participants did not receive any kind of financial compensation for participating in this study. Written informed consent was obtained from all subjects involved in the study.

Interviews were held by three trained health professionals external to the selected hospitals. Before the interviews, the coordinating team had meetings with hospital authorities to explain the objective of the study, introduce interviewers, and identify a suitable place to perform the interviews. Afterwards, hospital authorities sent reminders to identified hospital services to inform them about the study. Interviews were held in a quiet place, and participating maternity care providers were reminded about the objective of the study and that no identification was being recorded. Data were collected from December 2022 to May 2023.

We designed a semi-structured interview guide to explore four domains related to calcium supplementation during pregnancy: knowledge about calcium supplementation (specific information about the topic), attitudes towards calcium supplementation (ways of thinking, including stances, about the topic), current calcium supplementation recommendations (actual practices of health providers), and perceived barriers to and facilitators of calcium supplementation (see Supplementary Materials). Interviews were conducted until no significant new information was obtained from new participants. We confirmed data saturation after the last two interviews did not yield new insights within each of the sampled groups (such as midwives).

Interviews were transcribed verbatim and analyzed thematically using Atlas-Ti 24. The analysis followed the thematic framework of Clarke and Braun [14]. Briefly, this method consists of an iterative process that involves becoming familiar with the data, generating inductive codes, clustering them in themes, and locating examples [14]. We identified sub-themes inductively within each pre-defined domain (knowledge, attitudes, recommendations, barriers, and facilitators) based on the responses provided during the interviews. Two coders participated independently in coding the corpus for analysis. A matrix was created to outline the domains and to select illustrative quotes.

The study was approved by the Ethical Committee of the Buenos Aires province, resolution of September 2022 (PV-2022-29803007-GDEBA-DPEGSMSALGP).

## 3. Results

We interviewed a total of 74 maternity care providers: 17 (23%) were from Hospital A, 20 (27%) from Hospital B, and 37 (50%) from Hospital C. Table 1 presents the sociodemographic characteristics of the participants. The sample included midwives, gynecologists, obstetricians, nurses, and others (pediatricians, postgraduate medical residents, hospital health aide and caregivers and childcare workers).

### 3.1. Interviews

Maternity care providers usually referred to food and micronutrient intake in the general population, as well as iron and folic acid supplementation during pregnancy, when we asked about calcium supplementation during pregnancy. Gynecologists, obstetricians, and midwives spoke more about micronutrient physiology and nurses more about barriers to adhering to micronutrient supplementation, usually referring to iron and folic acid supplementation during pregnancy and calcium supplementation for the prevention of osteoporosis.

The sub-themes we identified from the interviews are given in Table 2.

### 3.2. Knowledge

We identified three sub-themes related to the maternity care providers’ knowledge about micronutrient supplementation during pregnancy.

Calcium intake is seen as relevant only for the prevention of osteoporosis. Calcium supplementation during pregnancy was considered relevant and associated with “strong bones” and “prevention of osteoporosis”. “Calcium is not only for pregnant women. Older people also need calcium to prevent osteoporosis. I take calcium” (nurse, 62 years old). “It is not something new. Calcium administration to pregnant women has been regulated in some way by scientific societies, for bone strengthening” (obstetrician–gynecologist, 61 years old).

Maternity care providers tended to report not knowing about the relationship between calcium supplementation and the prevention of preeclampsia. “I don’t know about the indication of supplements with just calcium. I recommend multivitamin supplements for pregnancy (…) supplement with all vitamins, calcium, magnesium, iron, and folic acid” (obstetrician–gynecologist, 43 years old).

When asked about calcium supplementation during pregnancy, maternity care providers stated that iron, folic acid, and multivitamin supplements are important during pregnancy and should be prioritized. They are seen as essential during pregnancy. Participants stressed the wide use of iron, folic acid, and multivitamins, but not calcium. Health providers noted that iron, folic acid, and multivitamin recommendations are widely endorsed, as opposed to calcium recommendations. “I never heard about calcium supplementation for blood pressure-related illnesses, to be frank. (…). I have heard more about other uses of calcium, or other minerals. And vitamins” (midwife, 58 years old).

Maternity care providers emphasized the importance of obtaining calcium through food, undermining the need for calcium supplements. They agreed that calcium-rich foods should be recommended to cover calcium requirements. They highlighted that most people know they need calcium. For them, prior knowledge is based on their own experience. “I know we need to eat calcium, but I don’t know anything more specific. We only need to drink milk and all those foods to get calcium” (nurse, 44 years old).

### 3.3. Attitude

We identified two sub-themes related to maternity care providers’ attitudes toward micronutrient supplementation during pregnancy. Overall, there was no homogeneous attitude regarding calcium supplementation and the reasons behind its use.

The first theme was related to prevention. Some maternity care providers considered that micronutrient supplementation should be undertaken regularly, as the population is at high risk of nutritional deficiencies. They linked micronutrient supplementation during pregnancy with a preventive practice for bone and dental health. “I prescribe supplements [in general] as a routine to all my population since they do not have resources or knowledge about calcium. They often cannot have an adequate diet as recommended” (obstetrician, 44 years old). Maternity care providers did not relate calcium supplementation to preeclampsia.

The second theme was related to treatment in cases of deficiency. Some maternity care providers considered supplementation is only necessary in cases of micronutrient deficiency, such as vitamin D deficiency. They pointed out the importance of requesting laboratory studies to support a clinical diagnosis and justify the use of micronutrient supplementation. “Personally, I do not prescribe iron to all my patients. I don’t think this is correct if the lab test doesn’t show a deficiency. That is not the same for folic acid, since the reason for its prescription is different” (obstetrician–gynecologist, 61 years old).

### 3.4. Recommendations

Most maternity care providers do not recommend calcium supplementation during pregnancy for the prevention of preeclampsia. We identified four different themes within this domain.

In the first theme, maternity care providers would recommend routine calcium supplementation, emphasizing the limited availability of resources for the population to maintain an adequate diet and the widespread malnutrition among them. In any case, the recommendation was not related to the prevention of preeclampsia (or high blood pressure). However, they mentioned that in the case of healthy pregnancies with adequate nutrition, the recommendation of supplements may be of little benefit. “Specific calcium supplementation, no. Maybe as part of a multivitamin/mineral supplement that contains calcium among other micronutrients” (obstetrician, 29 years old).

In the second theme, maternity care providers preferred a dietary approach by recommending consumption of calcium-rich foods. They emphasized the importance of promoting a balanced diet during pregnancy and raising awareness about the contribution of nutrients through foods. “I try to educate patients about nutrition to avoid ending up requiring not only supplementation but medicines. We know that hypertension, diabetes, and cholesterol are all preventable” (obstetrician–gynecologist, 39 years old). “I consider that sometimes a patient’s diet is deficient in calcium, but the patient doesn’t know alternatives to obtain calcium” (gynecologist, 56 years old).

In the third theme, maternity care providers prioritized iron and folic acid supplementation. Most maternity care providers agreed that supplementation with iron and folic acid is more important in this physiological period as they are essential due to the high incidence of anemia during pregnancy. “Only if the patient has chances to buy supplements, I recommend those supplements that have multivitamins. Otherwise, I only recommend iron and folic acid” (obstetrician, 46 years old).

Finally, in the fourth theme, maternity care providers recommended supplementation after a specific diagnosis of serum calcium deficiency, risk of osteopenia or osteoporosis, or in cases of hypoparathyroidism. “(Supplementation should happen only) in cases where serum values are low (…). And only if the diet is insufficient” (resident, 31 years old).

### 3.5. Barriers

Most maternity care providers reported that calcium supplementation during pregnancy is not prescribed and mentioned several barriers.

Many providers did not recommend calcium supplementation, as they emphasized calcium can be obtained through diet. “A good and rich diet can remedy most nutritional deficiencies, including calcium” (gynecologist, 33 years old). Other participants mentioned that supplements have low tolerance and prioritized the supplementation with other critical nutrients such as iron and folic acid. “Sometimes patients are intolerant to calcium pills” (obstetrician–gynecologist, 50 years).

Maternity care providers mentioned that they lack information and scientific evidence justifying the use of calcium supplementation during pregnancy. “Calcium supplementation during pregnancy is not implemented—it is still under discussion. I am not particularly informed on the topic” (obstetrician, 43 years old). Some participants considered that calcium supplementation is a new strategy, that the topic is still under discussion, that there is no evidence on the prevention of preeclampsia, and therefore it is not implemented on a regular basis like iron and folic acid. “We know that there is some deficiency (…), but there are no reports or statistics on calcium intake. There are studies and evidence on the need for iron supplementation (…). I have not received any statistics on calcium” (obstetrician, 47 years old). “Some time ago, calcium supplementation was debated, a gram a day, but afterwards there was no scientific evidence about it, so it is not used” (gynecologist, 39 years old). In addition to the deficient scientific evidence, the lack of local policy enforcement supporting calcium supplementation was frequently mentioned. “There is no legal framework to ensure access to calcium supplements” (gynecologist, 36 years old).

Participants also emphasized that there are no Ministry of Health guidelines and that there is no local training of health personnel to use calcium supplements during pregnancy. “It is never a priority, especially for these patients, who are young. Different for older women. And I have never heard about courses on this topic” (midwife, 58 years old).

Maternity care providers reported that calcium supplements are generally not available in hospitals. “Currently, calcium is not available (…), not even for prophylactic use in osteoporosis—there is none” (gynecologist, 33 years old). Participants linked the structural lack of resources with the fact that implementing calcium supplementation is not feasible. “Unless health authorities or a laboratory that provides free medicine, it’s very difficult that this population can access them” (obstetrician, 35 years old). “Cost, accessibility, for example. If it was available for free at primary health care level, where most patients go for pregnancy check-ups, it could be better, and if it was for free, even better” (obstetrician, 26 years old).

Finally, maternity care providers noted that the extremely vulnerable socioeconomic situation of the population restricts the recommendation of supplements. They agreed that, considering the economic status of the population, providers often refrain from prescribing items that patients may not be able to afford. Calcium supplementation was described, in some cases, as an expendable luxury. If hospitals lack basic resources and no calcium supplements are available, it will not be implemented. “There are other more important things to consider and deal with” (gynecologist, 45 years old). “In the hospital sometimes, patients must buy (supplements) themselves” (obstetrician–gynecologist, 50 years).

Despite several providers mentioning the importance of calcium supplementation for the prevention of osteoporosis, they were not aware of existing interventions to improve calcium intake. “I haven’t heard of calcium supplementation policies of any kind ever” (gynecologist, 39 years old).

### 3.6. Facilitators

Facilitators mentioned by maternity care providers were mainly related to institutional factors, yet access to information was mentioned as well. Maternity care providers described the availability of calcium supplements delivered free of charge in public hospitals as a fundamental facilitator of the implementation of calcium supplementation during pregnancy. “Delivering calcium supplements in hospitals, as iron and folic acid supplements” (nurse, 44 years old); “I know that a state generic drug is essential for all medications” (obstetrician, 56 years old). They also mentioned the importance of primary health-care level to guarantee access to supplements: “Improve the health-care system, have a good primary level (…), for obstetric check-ups” (obstetrician–gynecologist, 66 years). Access to information by users and maternity care providers was mentioned as another facilitator: “Information endorsed by medical athenaeums and clinical guidelines” (gynecologist, 55 years old); “information on the benefits of calcium” (nurse, 43 years old); “information, updates for both patients and us, about supplementation” (obstetrician–gynecologist, 38 years old).

## 4. Discussion

In this study, we showed that most maternity care providers reported being unaware of the recommendations of calcium supplementation during pregnancy for the prevention of preeclampsia. Most of them highlighted the importance of maintaining a healthy balanced diet during pregnancy including calcium-rich foods to cover pregnancy needs.

Maternity care providers acknowledged a deficient diet in the population; however, they perceived the socioeconomic situation as a barrier to recommending another micronutrient supplement such as calcium, which is not seen as important. Information related to local widespread calcium intake inadequacy and difficulties for the population to reach pregnancy dietary calcium recommendations through calcium-rich foods would be required to reinforce the need for strategies such as calcium supplementation in addition to nutritional education [15].

Calcium is not perceived as an essential micronutrient during pregnancy, as are iron and folic acid. The use of iron and folic supplements seems to be common and a well-accepted practice during pregnancy and could be used as an opportunity to include calcium. Maternity care providers reported that they would use calcium supplements during pregnancy if supported by scientific evidence [16,17]. Although the prescription of dietary supplement complexes containing calcium was reported, this was related to the perception of a poor diet in the general population rather than the prevention of preeclampsia.

Maternity care providers ´ training seems fundamental for the successful implementation of calcium supplementation. As preeclampsia does not seem to be a perceived problem in this population, training should include information regarding the local and global burden of HDP in pregnancy, including preeclampsia and maternal mortality data, as well as the impact that improving calcium intake could have in the population [11]. References to existing guidelines recommending calcium supplementation for populations with low calcium intake should be provided. However, the availability of local guidelines seems to be crucial to back up health providers’ actions [18,19]. Similar findings have been reported previously, highlighting the need for training of health-care providers, such as midwives, on the importance of calcium supplementation during pregnancy [20,21,22].

The availability of free calcium supplements, provided by the health system for prenatal care, seems to be a key issue in implementing calcium supplementation. Maternity care providers reported being cautious about prescribing medicines and supplements other than those distributed for free by health authorities, as they fear the population will not be able to afford them. Calcium is included in the list of essential national drug program of Argentina, REMEDIAR, but for other uses, such as osteoporosis and digestive problems [23]. Though REMEDIAR delivers free medicines through public health services, most maternity care providers agreed that there is a lack of calcium supplements at the primary health care level and in hospitals. Access to medicines also presents disparities in Argentina [24], especially for women [25].

Maternity care providers remarked there is no legal framework to prescribe calcium supplements during pregnancy, as local prenatal care guidelines only recommend iron and folic acid supplements [26]. However, if there were guidelines and training endorsed by the Ministry of Health, they would agree to recommend calcium supplements.

As shown in other studies, calcium intake is related to bone and dental health and calcium supplements to the prevention of osteoporosis in older adults, especially women [16]. Maternity care providers’ references to calcium supplementation were more related to their own experiences than to their practice and evidence. Most maternity care providers interviewed were women and aware of osteoporosis problems of women in older ages. Information about bone health and prevention of osteoporosis could be used to reinforce the need to improve calcium intake throughout pregnancy.

Future studies should assess the perceptions of pregnant women and policymakers regarding calcium supplementation during pregnancy. It is also crucial to gather information about the local infrastructure required to provide free calcium supplements in antenatal care settings. Conducting an economic evaluation with local data could inform policymakers about the necessity of implementing strategies to reduce preeclampsia. Confidence in such efforts among governing health actors would likely increase with collaborative coordination.

### Limitations

The present study has certain limitations. Firstly, our selection of hospitals was by convenience and together with the sample strategy limits the generalizability of the results beyond our specific study sample. However, our findings can still offer valuable insights for designing strategies to implement calcium supplementation in antenatal care within similar settings. Secondly, participants may have felt hesitant to express their thoughts and opinions due to the recruitment procedure. Nevertheless, the emergence of similar topics across the three hospitals indicates a consistent pattern in responses. Thirdly, using a semi-structured interview guide may have resulted in overlooking certain topics not addressed in the conversations. However, this approach allowed us to conduct a detailed analysis of the research questions at hand. Finally, our study focuses on the overall responses given by maternity care providers, rather than distinguishing between the different roles they play in providing care during pregnancy. Despite these limitations, the relative scarcity of formative studies on calcium implementation in Latin America underscores the importance of our study and highlights the need for future investigations. Furthermore, despite these limitations, our study provides essential data for evaluating the scalability of a calcium supplementation policy in Buenos Aires and offers insights into the current knowledge, attitudes, and perceptions of maternity care providers. This information is crucial for guiding future interventions and policy.

## 5. Conclusions

This study underscores the connection between maternity care providers’ knowledge, attitudes, and practices regarding calcium supplementation during pregnancy and the barriers to implementing calcium supplementation policies in Metropolitan Buenos Aires, Argentina. The government’s free provision of calcium supplements, coupled with training that reinforces the scientific evidence supporting their use to prevent preeclampsia alongside documented recommendations from credible sources, is crucial to ensure maternity care providers adopt the use of calcium supplements in antenatal care. Furthermore, developing calcium-related messaging to communicate its importance in preventing preeclampsia is fundamental to overcoming maternity care providers’ reservations. These findings can guide the development of evidence-based materials for nutrition education interventions and knowledge translation initiatives.

## Figures and Tables

**Table 1:** Sociodemographic characteristics of maternity care providers interviewed in three hospitals of Metropolitan Buenos Aires (*n* = 74).

Characteristics	Hospital A		Hospital B		Hospital C		Total	
	*n*	%	*n*	%	*n*	%	*n*	%
Age (years)								
<40	6	30.0	6	35.3	25	67.6	37	50
≥40	14	70.0	11	64.7	12	32.4	37	50
Sex								
Female	19	95.0	16	94.1	32	86.5	67	90.5
Male	1	5.0	1	5.9	5	13.5	7	9.5
Profession								
Midwives	11	55.0	6	35.3	2	5.4	19	25.7
Obstetricians and gynecologists	5	25.0	8	47.1	7	18.9	20	27.0
Nurses	4	20.0	2	11.7	15	40.5	21	28.4
Other *	0	0	1	5.9	13	35.2	14	18.9
Years working at the hospital								
<1	2	10.0	1	5.9	8	21.6	11	14.9
≥1	18	90.0	16	94.1	29	78.4	63	85.1

* Pediatricians, postgraduate medical residents, hospital health aides, caregivers, and childcare workers.

**Table 2:** Pre-defined and emergent themes yielded in the analysis.

**Pre-Defined Themes**	**Emergent Themes**
Knowledge	Calcium intake during pregnancy for the prevention of osteoporosis Supplementation during pregnancy including iron, folic acid, and multivitamins Importance of calcium intake through foods
Attitudes	Calcium supplementation for osteoporosis prevention Calcium supplementation to treat calcium-deficient individuals
Recommendations	Agree with routine calcium supplementation. Supplements with only calcium are currently not prescribed Preference for food recommendations to improve calcium intake Recommendations should focus on iron and folic acid supplementation Calcium supplementation should be recommended only after a diagnosis of calcium deficiency
Barriers	No need to supplement as calcium is accessible through diet Low tolerance to calcium supplements Priorities should be given to iron and folic acid supplementation Lack of scientific evidence of calcium supplementation Calcium supplementation is seen as a new untested strategy to address preeclampsia Lack of local policy enforcement and guidelines to recommend calcium supplementation Lack of health professional training to implement calcium supplementation during pregnancy Calcium supplements are not available at primary health care centers or in hospitals Socioeconomic situation of the population makes it inaccessible High costs would be transferred to the patients Lack of knowledge of current calcium implementation policies
Facilitators	Free calcium supplement distribution Calcium supplements available at the primary care level Access to information for users and maternity care providers.

## Data Availability

The data presented in this study are available on request from the corresponding author due to privacy reasons.

## Supplementary Materials

The following supporting information can be downloaded at https://www.mdpi.com/article/10.3390/nu16162734/s1. Table S1: Participant interview guide.
