# Supplementary material for: Knowledge, Attitudes, and Perceptions of Maternity Care Providers on the Implementation of Calcium Supplementation during Pregnancy in Three Public Hospitals in Argentina: A Qualitative Study
Source: Nutrients. 2024 Aug 16;16(16):2734. doi: 10.3390/nu16162734 (PMC11356881; doi:10.3390/nu16162734)
Supplement: Supplementary file 1 [file nutrients-16-02734-s001.zip › nutrients-3094374-supplementary.pdf]

Table S1: Participant interview guide.

SEMI-STRUCTURED INTERVIEW	GUIÓN SEMIESTRUCUTRADO DE ENTREVISTA (Spanish version)
**1 Socio-demographic data**<br>1.1 How old are you?<br>1.2 What is your profession/specialty?<br>1.3 Where do you work?	**1. Datos demográficos**<br>1.1 ¿Qué edad tiene?<br>1.2 ¿Cuál es su profesión/especialidad?<br>1.3 ¿En dónde trabaja?
**2 Knowledge about calcium, supplementation and pregnancy**<br>2.1 Could you tell me what you know about calcium supplementation?<br>2.2 Could you tell me what you know about the link between calcium and blood pressure?<br>2.3 Could you tell me what you know about the link between calcium and blood pressure in pregnant women?<br>2.4 Do you know any guidelines or recommendations on calcium supplementation?	**2. Conocimientos sobre calcio, suplementación y gestación**<br>2.1. ¿Podría comentarme qué sabe sobre la suplementación de calcio?<br>2.2. ¿Podría comentarme qué sabe sobre el vínculo entre calcio y presión arterial?<br>2.3. ¿Podría comentarme qué sabe sobre el vínculo entre calcio y presión arterial en personas gestantes?<br>2.4. ¿Conoce guías o recomendaciones sobre la suplementación de calcio?
**3. Attitudes about calcium, supplementation and pregnancy**<br>3.1. How do you feel about vitamin and mineral supplementation in general?<br>3.2. In particular, what do you think about calcium supplementation?<br>3.3. Do you recommend calcium supplementation?<br>3.4. Do you know what your colleagues think about this issue?	**3. Actitudes sobre calcio, suplementación y gestación**<br>3.1. ¿Qué opina, en general, sobre la suplementación de vitaminas y minerales?<br>3.2. ¿Y en particular qué opina sobre la suplementación de calcio?<br>3.3. ¿Usted recomienda la suplementación de calcio?<br>3.4. ¿Sabe qué opinión tienen sus colegas sobre este tema?
**4. Barriers and facilitators**<br>4.1. What aspects imply potential or effective barriers to supplementation?<br>4.2. What aspects imply potential or effective facilitators for supplementation?	**4. Barreras y facilitadores**<br>4.1. ¿Qué aspectos implican barreras potenciales o efectivas para la suplementación?<br>4.2. ¿Qué aspectos implican facilitadores potenciales o efectivos para la suplementación?
**5. Wrap-up**<br>5.1. Finally, I would like to know if there is any topic that you consider relevant, and we have not discussed.<br>5.2. Do you have any closing questions or comments?<br>5.3. Would you like to receive the results of the study once completed?	**5. Cierre**<br>5.1. Por último, me gustaría saber si existe algún tema que no hayamos tocado y que considere relevante.<br>5.2. ¿Tiene alguna pregunta o comentario de cierre?<br>5.3. ¿Le gustaría que le compartamos los resultados del estudio una vez finalizado?
**Thank you very much**	**Muchas gracias**
